# Does scent attractiveness reveal women's ovulatory timing? Evidence from signal detection analyses and endocrine predictors of odour attractiveness

**DOI:** 10.1098/rspb.2022.0026

**Published:** 2022-03-09

**Authors:** Mei Mei, Rachel L. Grillot, Craig K. Abbey, Melissa Emery Thompson, James R. Roney

**Affiliations:** ^1^ Department of Psychological and Brain Sciences, University of California, Santa Barbara, CA, USA; ^2^ Department of Anthropology, University of New Mexico, Albuquerque, NM, USA

**Keywords:** scent attractiveness, concealed ovulation, oestradiol, progesterone, human mating

## Abstract

Odour cues associated with shifts in ovarian hormones indicate ovulatory timing in females of many nonhuman species. Although prior evidence supports women's body odours smelling more attractive on days when conception is possible, that research has left ambiguous how diagnostic of ovulatory timing odour cues are, as well as whether shifts in odour attractiveness are correlated with shifts in ovarian hormones. Here, 46 women each provided six overnight scent and corresponding day saliva samples spaced five days apart, and completed luteinizing hormone tests to determine ovulatory timing. Scent samples collected near ovulation were rated more attractive, on average, relative to samples from the same women collected on other days. Importantly, however, signal detection analyses showed that rater discrimination of fertile window timing from odour attractiveness ratings was very poor. Within-women shifts in salivary oestradiol and progesterone were not significantly associated with within-women shifts in odour attractiveness. Between-women, mean oestradiol was positively associated with mean odour attractiveness. Our findings suggest that raters cannot reliably detect women's ovulatory timing from their scent attractiveness. The between-women effect of oestradiol raises the possibility that women's scents provide information about overall cycle fecundity, though further research is necessary to rigorously investigate this possibility.

## 1. Introduction

Concealed ovulatory timing plays an important role in major theories of the evolution of human pair bonding [1,2], with pair bonding, in turn, having contributed to the evolution of greater human brain size and intelligence [3]. Yet, research suggests that human ovulatory timing may not be entirely concealed, since stimuli from women are rated as more attractive near ovulation than at other times of the cycle ([4]; cf. [5]). Thus, a tension exists between arguments for the evolution of human pair bonding and empirical research examining cues of ovulatory timing. This has left unresolved an important question for understanding human evolution: is women's ovulatory timing effectively concealed?

Odour cues play especially important roles in revealing ovulatory timing in nonhuman mammals. Males in many species strongly prefer natural scents collected from females during cycle phases when conception is possible [6], and in some species such cues are *necessary* for males' pursuit of females since disruption of scent cues abolishes sexual interest in fecund and receptive females (e.g. [7,8]). Ovarian hormones associated with temporal shifts in fecundity regulate these cycle phase shifts in odour attractiveness: oestradiol administration restores scent attractiveness in gonadectomized females of multiple species [9–11], whereas progesterone administration reduces odour attractiveness [9,11,12]. Since a number of these hormone effects occur in nonhuman primates [9,12], these data provide an important phylogenetic background against which to compare the possible endocrine correlates of scent attractiveness in humans.

Women's odour samples collected during the putative fertile window (i.e. the cycle days when conception is possible) have on average been rated as more attractive than those collected during the non-fecund luteal phase ([13–17]; cf. [18]). Although some authors have concluded from these findings that human ovulation is not actually concealed (e.g. [15,16]), there are significant questions regarding whether the scent attractiveness shifts are reliably diagnostic of fertile window timing. Havlicek *et al*. [14], for instance, found in their sample that between-women variability in women's odour attractiveness was much larger than within-cycle variability, such that some women smelled better outside of the fertile window than other women did inside of it. Such patterns could be consistent with effectively concealed ovulatory timing even if, on average, women smell more attractive during the fertile window [19]. In the current research, for the first time, we apply signal detection theory to more formally assess whether women's fertile window timing is detectable from cycle phase shifts in their odour attractiveness. Signal detection theory provides a rigorous quantitative approach to assessing the accuracy of decision-making; in this case, the accuracy of inferring fertile window timing from odour attractiveness.

**Figure 1.**
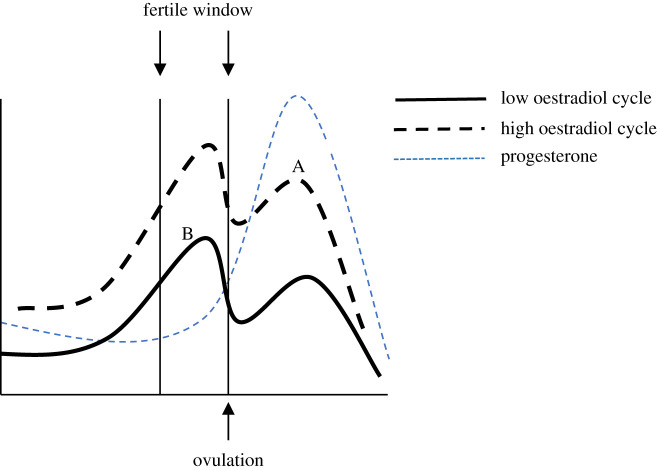
Prototypical hormone secretions across human ovulatory menstrual cycles. The two oestradiol curves represent different cycles that vary in oestradiol production. A single progesterone curve is depicted for simplicity.

Endocrine predictors of odour attractiveness carry important implications for the detectability of women's ovulatory timing and can be used to assess evolutionary changes in reproductive signalling relative to nonhuman mammals. Figure 1 depicts prototypical patterns of oestradiol and progesterone production across ovulatory human menstrual cycles. It can be seen from the figure that positive effects of oestradiol combined with negative effects of progesterone on odour attractiveness, as seen in many nonhuman species, could produce odour attractiveness peaks that are tightly coupled to the fertile window. Thus, if ovulatory timing is detectable via odour cues in humans, one would expect conservation of these two hormonal correlates of scent attractiveness.

If, however, women's scent attractiveness tracks fluctuations in oestradiol but not progesterone, then cues of ovulation may be sufficiently suppressed that perceivers do not get reliably diagnostic information regarding fertile window timing. This follows from between-women and between-cycle variability in oestradiol, as oestradiol tends to be elevated across the cycle in cycles with higher conception probabilities [20]. In figure 1, oestradiol is higher during the luteal phase of the higher oestradiol cycle (point A) than it is in the fertile window of the lower oestradiol cycle (point B); if odour attractiveness tracks oestradiol only, then it will be greater at point A than at point B, which is inconsistent with odours acting as clear cues of ovulatory timing. Note that if progesterone strongly reduces odour attractiveness, then fertile window samples will consistently smell better than luteal phase samples regardless of between-cycle variability in oestradiol. Thus, one evolutionary pathway for concealing ovulatory timing might have entailed suppressing the effects of progesterone on body odours [21]. Perceivers in that case may have maintained preferences for odours associated with higher oestradiol because elevated oestradiol signals that a woman is experiencing higher fertility cycles in general (see [19,22]).

These considerations suggest that endocrine predictors of odour attractiveness can provide important clues regarding whether and how human ovulation became concealed. One prior study collected an odour sample from women near ovulation and reported that, between-women, oestradiol was positively and progesterone negatively associated with ratings of odour attractiveness [23]. This between-women comparison does not directly assess whether elevated luteal phase progesterone suppresses odour attractiveness relative to other days within the same cycle, however, and in fact no prior studies appear to have tested the relationship between within-women shifts in ovarian hormones and within-women changes in odour attractiveness. The present study was designed in part to address this gap in the human literature.

Here, women collected overnight odour samples once every five days for 30 days, as well as saliva samples on corresponding days, from which oestradiol and progesterone were assayed. In addition, urinary luteinizing hormone (LH) tests were administered to estimate ovulatory timing.

There were three main aims for the study. (i) To test whether samples collected during the fertile window smell more attractive than those collected on other cycle days when sampling occurs across the entire cycle. Based on prior findings, we predicted greater attractiveness ratings for fertile window odour samples. (ii) To assess whether odour attractiveness is diagnostic of fertile window timing using signal detection analyses. Although we did not formulate a precise prediction for this aim, based on the expectation that ovulatory timing is concealed, we anticipated that the overlap in attractiveness ratings between fertile window and other samples would be too great for fertile window timing to be accurately diagnosed from odours. (iii) To test the hormonal correlates of women's odour attractiveness. We predicted positive associations between oestradiol concentrations and odour attractiveness—both within-cycles and between-women—but null associations between progesterone and odour attractiveness. This pattern could reconcile within-women ovulatory shifts in odour attractiveness with effectively concealed ovulatory timing: in figure 1, for instance, if only oestradiol affects odour attractiveness, its positive effects should produce small within-cycle shifts in attractiveness but with fertile window timing effectively obscured by the between-cycle variability in this hormone. Null effects for progesterone, if supported, would provide preliminary evidence for the suppression of progesterone-related cues as a proximate mechanism for the evolution of concealed ovulatory timing in humans.

## 2. Material and methods

### (a) Participants

Women odour sample donors were undergraduate students recruited conditional on no pregnancy, lactation, or use of any hormonal contraceptives within the last six months. A total of 52 women began the 30-day study, but six dropped out early in the data collection, leaving a sample of 46 women with both salivary hormone assays and odour samples. Mean age of these women was 20.24 ± 0.16 years. Twenty-two women self-reported Asian ethnicity, 11 White, 10 Latinx, and 3 other; ethnicity was unrelated to odour attractiveness and is not considered further. Women participants were paid up to $180 US for full completion of all study procedures (including participation in laboratory sessions unrelated to the current research), and lower, pro-rated amounts in cases with missing samples. Sample size was the largest that our budget for subject payments and hormone assays could support.

Scent raters were male undergraduate students. Due to a clerical error, their ages and ethnicities were not queried, but should be similar to the women donors given that they attended the same university. Among 66 original raters, data from five men who indicated a gay sexual orientation were excluded (since the raters rated women's odours for sexiness), leaving a final sample of 61 men. Raters received either $10 US for their participation, or partial fulfilment of course requirements.

### (b) Donor materials and procedures

Women participants first attended an orientation session for study instructions and distribution of odour collection materials. Data collection began at random times in the menstrual cycle. Women collected daily urinary LH tests (Babi One Step Urine Ovulation Test) in the afternoon starting on any days on which they were not menstruating, and uploaded photos of the test results on a secure website; we contacted the women and instructed them to stop collecting LH tests within a given cycle after a positive test result was followed by at least three consecutive days of negative results. Women also completed a daily survey on a secure website each morning in which they reported feelings and behaviours on the prior day (those variables are not analysed here), and whether they menstruated on the day in question.

At the beginning of the study and once every five days afterwards, women were instructed to collect odour samples and a saliva sample. Saliva samples were scheduled for morning collection at least 30 min after any eating or drinking, and before any teeth brushing. Women were instructed to rinse with clean water a few minutes before depositing 1.5 ml of saliva into prelabelled polypropylene vials via passive drool. Photos of the saliva samples were uploaded to the daily survey website, and the samples were then stored within home freezers until being delivered to our laboratory when women attended laboratory sessions at one- to two-week intervals (samples were then stored at −40°C until shipping for assay). On the same days as saliva collection, women were instructed to prepare for odour sample collection by showering with unscented soap and refraining from smoking, drinking alcohol, sexual activity, wearing artificial fragrances, and sleeping with pets or other people on the night of sample collection. Before going to sleep, they affixed cotton pads under each armpit using athletic tape, and also placed a pantyliner into their underwear for collection of vaginal odours (this step was omitted during menstruation); a clean, unscented T-shirt was then worn over their torsos. Samples were worn overnight while sleeping, and upon waking were placed into pre-labelled freezer bags and stored in home freezers until delivery to our laboratory (samples were then stored at −40°C until their use in rating sessions). All necessary collection materials were provided to participants (including unscented soap and clean T-shirts), and shirts were laundered by the investigators with unscented detergent before their use. Data collection continued for 30 days, such that women provided up to six pairs of odour and corresponding day saliva samples.

### (c) Rating materials and procedures

To avoid rater fatigue, five different rating sessions were organized in which 10–17 raters rated the odours of 9–10 women per session. Only the underarm samples were rated in this study, with cotton pads placed into pre-labelled, individual glass jars and thawed for 3 h before the start of a session in which they were used. For each sample collection day, the left or right arm pad was chosen randomly and placed into a separate glass jar, such that six odour samples from each woman (two women had four samples due to missing sample days) were presented for rating. Samples from the same women were grouped together into rating stations, such that a rater would rate all of the samples from one woman consecutively before moving on to the samples from another woman. Latin squares were used to counterbalance the ordinal rating order across stations and also the order of samples rated within each station.

Male raters were informed via a consent form that they would be rating women's body odours. Each rater was given a rating sheet that contained an individual, prespecified rating order as determined by the Latin squares. For each sample, men were instructed to open the lid of the jar, take a deep sniff, close the lid, and write their ratings on the paper rating form. Samples were rated for pleasantness, sexiness, and intensity on 7-point Likert scales. After rating samples, raters completed a brief survey regarding their sexual orientation and relationship and sexual history, as well as questions about their sense of smell: all raters reported being non-smokers and none reported deficits in scent perception.

### (d) Hormone assays

Saliva samples were shipped on dry ice to the Comparative Human and Primate Physiology Center at the University of New Mexico. Samples were thawed, vortexed, and centrifuged for 15 min prior to assay to break up and precipitate mucins. Oestradiol and progesterone were assayed using kits designed for saliva by Salimetrics (State College, PA). Oestradiol had a least detectable dose of 0.1 pg ml^−1^ and progesterone a least detectable dose of 5 pg ml^−1^; samples below these thresholds were set to the respective detection limits. Interassay CVs were 3.4% (low control) and 3.6% (high control) for oestradiol, and 7.5% (low) and 6.5% (high) for progesterone. Intraassay CVs were 5.4% and 6.0% for oestradiol and progesterone, respectively. As checks on the validity of the hormone assays, graphs of oestradiol and progesterone concentrations as functions of cycle day are presented in the electronic supplementary material.

### (e) Cycle phase estimation

Urinary LH tests reliably indicate ovulation within about 24–36 h from the first positive test within a given cycle [24,25]. As such, we estimated the day of ovulation in each cycle as one day after the first positive LH test result as judged from the test photos uploaded by women donors, and checked that these days preceded elevations in progesterone measured from saliva samples. We were able to identify a day of ovulation for 32 women with this method. For 14 women, a day of ovulation could not be clearly estimated: six of these women had no positive LH test results, five women had sporadic partial test signals and no elevations in progesterone, and three women had multiple positive LH signals that were sporadically scattered throughout cycle days but without any clear elevation in progesterone. For cycles with an estimated day of ovulation, the fertile window was defined as the day of ovulation and the preceding five days [26], and a binary variable was created with sample days within this window coded as one and outside this window coded as zero. We also computed a more continuous conception risk variable that assigned specific conception risk values to individual days within the fertile window (these values were drawn from those reported in [27]), but with days outside the fertile window still assigned conception risk of zero (see electronic supplementary material for further description of this variable). Because women began data collection at random points in their cycles, in some cases a day of ovulation was detected in one cycle but samples were also collected in the other cycle for which ovulatory timing (or anovulation) was unknown. Our primary fertile window analyses used only sample days from which we could assign a specific cycle day relative to an estimated day of ovulation; however, in some cases, samples from a different cycle were still likely to be outside the fertile window for that cycle (e.g. near the end or start of a cycle), and in electronic supplementary material we present analyses using a fertile window variable with such days coded as zero.

### (f) Statistical analyses

As a general analysis strategy, we followed Gildersleeve *et al*. [13] who used a similar scent rating design, and employed cross-classified multi-level regression analyses on scent ratings with random error terms included for both rater and donor intercepts and slopes. Because pleasantness and sexiness ratings were highly correlated (*r* = 0.75 across all ratings), we used the mean of these ratings as our measure of scent attractiveness. These mean values were grand-mean standardized in order to make effect sizes interpretable in s.d. units. To help assess the robustness of parameter estimates, we also computed bootstrapped 95% confidence intervals for each estimate (by drawing 1000 samples of donor subjects for each model using the stratified bootstrapping function in SPSS v. 26), and report these in addition to the more conventional *p*-values.

For our first aim of assessing fertile window associations with odour attractiveness, the fertile window variables were entered as binary predictors of scent ratings in the multi-level models. Parameter estimates in these models denote the change in odour ratings from outside to inside the fertile window. The analysis of fertile window timing as a binary predictor was planned before our data collection and is thus the primary analysis presented in the main text; in electronic supplementary material, we present exploratory analyses that assign different conception risk values to days within the fertile window. All fertile window analyses were restricted to those scent donors for whom a day of ovulation could be estimated.

For our second aim, we employed receiver operating characteristic (ROC) analyses to assess whether raters were able to reliably discriminate fertile window samples. The ROC curve plots the ‘hit’ rate—the probability that a fertile window sample is rated above a given attractiveness rating threshold—against the ‘false alarm’ rate—the probability that a non-fertile window sample is rated above the same threshold. Plotting these values against the full range of rating thresholds generates an empirical ROC curve. Area under the curve (AUC) was then employed as a common measure of discrimination performance: perfect performance produces an AUC of 1, chance performance an AUC of 0.5, with ‘moderate’ performance often associated with AUCs of at least 0.75 in fields such as medical imaging [28,29]. To test raters' ability to discriminate fertile window from other samples within the same women, AUC values were computed for each donor and rater combination; these values were then treated as the dependent variable in a multi-level regression model (with random intercept terms for donors and raters) that tested whether the grand-mean intercept AUC value differed from 0.5. To test whether raters could discriminate the fertile window across different women as well as within-women, a single ROC curve was computed for each rater based on all of their ratings and the corresponding AUC values were tested for difference from 0.5.

For our third aim of assessing hormonal predictors of odour attractiveness, hormone concentrations on odour sampling days were entered as predictors in the multi-level regression models predicting attractiveness ratings. A mixed regression model revealed that salivary oestradiol concentrations were associated with time of day that samples were collected (*γ* = 0.04, *p* = 0.005), and so standardized residuals from time of day were computed for oestradiol. Progesterone was not associated with time and so grand-mean standardized values were first computed. The standardized values for both hormones were next centred within-women for use as Level-1 predictors in the regression models: parameter estimates for these models thus depict the change in standardized odour ratings associated with a within-woman 1 s.d. change in hormones assessed in grand-mean s.d. units. (Because predictor variables and the dependent variable were standardized, regression coefficients in these models can be interpreted similar to standardized beta coefficients.) Level-2 hormone variables were mean hormone values for each woman, also in grand-mean s.d. units. To assess the strength of evidence for the predicted null effect of progesterone, we computed a Bayes factor for this variable by comparing Bayesian Information Criteria (BIC) statistics across alternative models using the computations specified in [30].

## 3. Results

### (a) Fertile window associations with odour attractiveness

**Figure 2.**
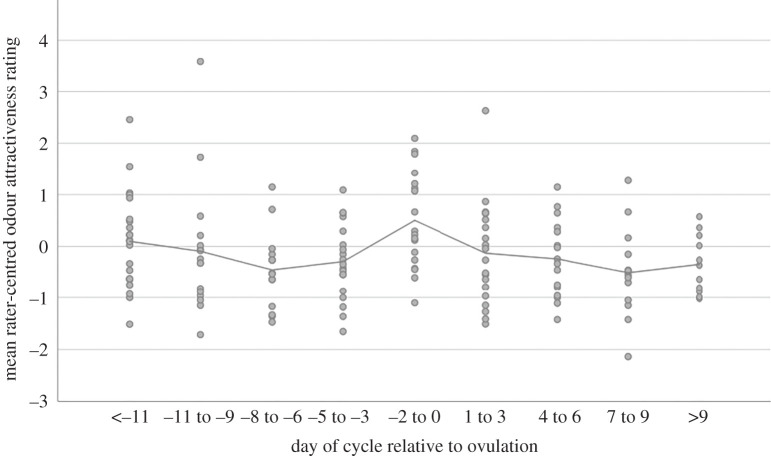
Mean odour attractiveness ratings across cycle days. To remove rater differences in scale usage for the figure, ratings were first centred by subtracting each rater's mean rating from their individual sample ratings, and these rater-centred values were then grand-mean standardized. Each data point represents the mean rating for an individual odour sample. Cycle days are placed into 3-day bins and the trend line plots the mean value for each bin.

Figure 2 presents grand-mean standardized odour attractiveness ratings by region of the cycle. Fertile window samples include those collected between days −5 and 0, where day 0 is the estimated day of ovulation. A mixed regression model comparing those days to the other sample days within-women produced evidence for a positive effect of fertile window timing on odour attractiveness: *γ* = 0.13, *p* = 0.04, CI: [0.04, 0.21]. Data in figure 2, however, suggest that odour attractiveness may increase only in the late fertile window. When we excluded days −2 to 0 from the analyses, fertile window timing no longer predicted odour attractiveness: *γ* = 0.03, *p* = 0.70, CI: [−0.11, 0.11], suggesting no elevation of scent attractiveness in the early fertile window. By contrast, when we constructed a late fertile window variable that coded days −2 to 0 as 1 and all other cycle days as 0, odour attractiveness ratings were higher during the late fertile window relative to other cycle days from the same women: *γ* = 0.27, *p* = 0.002, CI: [0.13, 0.37]. By contrast to odour attractiveness ratings, fertile window timing was not associated with ratings of odour intensity, for either the full or late fertile window (*p*s > 0.20). Analyses presented in electronic supplementary material demonstrate similar results when a more continuous measure of conception risk was employed and when sample days from adjacent cycles that were unlikely to be in the fertile window were coded as 0 for fertile window timing rather than being coded as missing.

### (b) Detectability of fertile window timing

**Figure 3.**
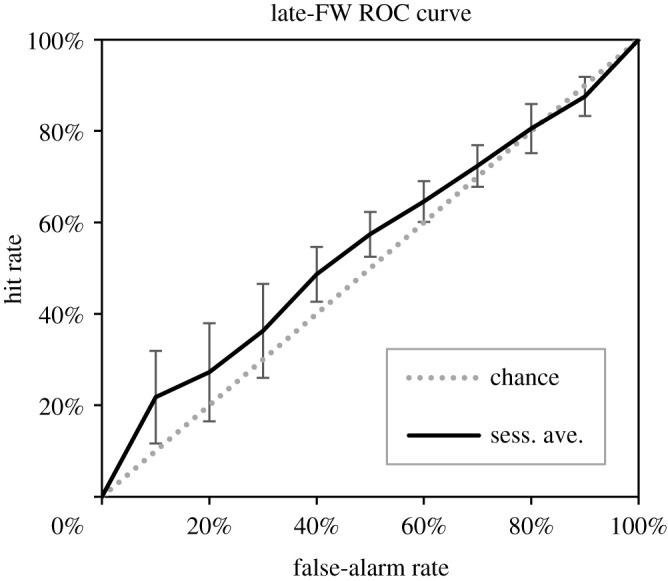
Discrimination performance for late fertile window data. The average ROC curve, evaluated at 10% false-alarm rate intervals is plotted along with error bars representing 95% confidence intervals on the associated hit rates. Sess. Ave. indicates the average hit rate within rating sessions. For reference, the diagonal line representing chance performance is also plotted.

Because the above analyses suggested effects on odour attractiveness only in the late fertile window, we first computed ROC curves for each rater by donor combination for the late fertile window variable. Figure 3 depicts the average ROC curve. A multi-level regression model treating each rater by donor AUC as the dependent variable with random intercepts for raters and donors estimated a grand-mean intercept AUC of 0.57, CI: [0.50, 0.64], *t*_17.99_ = 2.11, *p* = 0.049. Thus, although this is a very low AUC value, it was above the chance value of 0.50. The random effect of raters on the AUC values was not significant (Wald *Z* = 0.75, *p* = 0.45), suggesting that raters did not differ in their ability to discriminate fertile window samples. There was marginal evidence for a random effect of donors on AUC values (Wald *Z* = 1.73, *p* = 0.08).

The ROC analyses above tested raters’ ability to discriminate the fertile window when comparing across samples within-women. When ROC curves were instead constructed for each rater by comparing ratings for late fertile window versus other samples across all of the stimuli that each man rated, discrimination performance was lower: the mean AUC across raters was 0.53, CI: [0.49, 0.57], which did not differ significantly from 0.50 (1-sample *t*_50_ = 1.31, *p* = 0.20). By focusing on the late fertile window, we attempted to provide the best chance for ROC analyses to discover strong discrimination of fertile window timing. Analyses in electronic supplementary material show that discrimination performance was even weaker when we analysed the full fertile window.

### (c) Hormonal predictors of odour attractiveness

We predicted positive effects of oestradiol on odour attractiveness when considering both within-women (Level-1) and between-women (Level-2) effects, but null effects of progesterone. Table 1 presents a model that tests simultaneous main effects of oestradiol and progesterone on odour attractiveness ratings. We found evidence only for a positive correlation between mean oestradiol and mean odour attractiveness (the Level-2 oestradiol effect in table 1). Contrary to our prediction, odour samples from the same women did not smell better on days when their oestradiol was higher. There were no significant interactions between oestradiol and progesterone (at Level-1 or Level-2) when interaction terms were added to the model in table 1 (*p*s > 0.20). Statistical conclusions for zero-order effects of the hormones matched those from the multiple regression presented in table 1, with evidence supporting only a positive Level-2 effect of oestradiol: *γ* = 0.44, *p* = 0.04, CI: [0.30, 0.59].

**Table 1.** Multi-level regression model testing the simultaneous associations of oestradiol and progesterone with grand-mean standardized odour attractiveness ratings.

predictor	estimate (d.f.)	*p*-value	bootstrapped 95% CI
Level-1 oestradiol	0.02 (45.44)	0.57	−0.02, 0.07
Level-1 progesterone	−0.001 (31.85)	0.98	−0.06, 0.06
Level-2 oestradiol	0.49 (48.71)	0.06	0.27, 0.66
Level-2 progesterone	−0.03 (38.22)	0.68	−0.08, 0.03

Null effects for progesterone were consistent with our expectations. We estimated a Bayes factor for the within-women effect of progesterone by comparing BIC values for a model with oestradiol effects only to one in which the fixed effect of Level-1 progesterone was added. The Bayes factor suggested that the observed data were 10.73 times more likely under a model without the progesterone effect compared to one with it. Likewise, comparing a model that adds the Level-1 progesterone effect to a null model with random intercepts produced a Bayes factor suggesting that the data were 15.32 times more likely under the null model.

We assessed models that included both a binary fertile window variable and the Level-1 hormonal predictors of odour attractiveness for the subset of women for whom we could estimate the day of ovulation. For days without missing hormone data, the zero-order late fertile window effect was: *γ* = 0.21, *p* = 0.007, CI: [0.07, 0.32]. Addition of Level-1 oestradiol and progesterone as predictors had a negligible effect on the fertile window effect size (*γ* = 0.19, *p* = 0.02, CI: [0.02, 0.31]), and BIC values suggested that addition of the hormone terms harmed the fit of the model. Thus, although there was a positive effect of late fertile window timing on odour attractiveness ratings, we found no evidence for hormonal mediation of this effect.

Finally, exploratory analyses revealed that women who self-reported being in a romantic relationship (*n* = 20) were rated as smelling more attractive, on average, than were single women (*n* = 26): *γ* = 0.17, *p* = 0.03, CI: [0.12, 0.22]. This effect persisted when relationship status was added to the hormone model in table 1, and by using BIC values to compare models, the best-fitting model contained only two Level-2 predictors: relationship status (*γ* = 0.17, *p* = 0.02, CI: [0.12, 0.22]) and mean oestradiol (*γ* = 0.42, *p* = 0.03, CI: [0.29, 0.58]).

## 4. Discussion

### (a) Fertile window associations with odour attractiveness

Our results corroborate prior evidence that women's odours smell more attractive, on average, during the fertile window relative to other cycle days. Furthermore, the data suggest that this effect is confined to the highest fecundity days (see [26]) during the late fertile window, with an effect size comparable to a standardized beta of 0.27. This pattern is remarkably similar to that reported by Gildersleeve *et al.* [13], who compared attractiveness ratings for two axillary samples per woman—one in the fertile window and one in the luteal phase—and reported that the fertile window advantage in attractiveness was larger for women whose high fertility sample was collected closer to ovulation. That study and the current one are the only two investigations of this question that have confirmed ovulatory timing via LH tests, and results from both converged on the conclusion that odour samples collected close to ovulation receive elevated mean attractiveness ratings.

### (b) Detectability of fertile window timing

Mean attractiveness ratings that are elevated in the fertile window do not necessarily entail that fertile window stimuli are clearly discriminable from stimuli produced at other times. Indeed, the ROC analyses presented here show very poor discrimination performance by raters. Even the best performance, depicted in figure 3, was associated with a mean AUC of 0.57, while Roe & Metz [28] assigned labels of ‘high,’ ‘medium,’ and ‘low’ discrimination performance to AUC values of 0.96, 0.86, and 0.70, respectively. Thus, although late fertile window samples do receive higher odour attractiveness ratings on average, there is sufficient overlap with the ratings from non-fertile window samples that discriminability is very poor. Figure 3 essentially shows that for any attractiveness threshold at which perceivers might implicitly categorize a sample as being in the fertile window and thus alter their behaviour in response to that sample (e.g. initiating courtship, sexual or mate guarding behaviours), the rate of false alarms to non-fertile window samples is nearly the same as the hit rate for the actual fertile window samples. As such, odour cues do not appear to provide diagnostic information regarding human ovulatory timing.

The ROC analyses were constructed in two ways to test different extant arguments regarding how perceivers might use odour information. In one set of analyses, a single ROC curve was constructed for each rater, thus comparing their performance for fertile window versus other samples regardless of whether samples were from the same or different women. This analysis is most relevant to claims that men might use odour cues to identify which women are currently fecund among different women and then use that information to target short-term mating effort [16]. However, the mean AUC in these analyses showed that discrimination performance was not better than chance. Thus, we found no evidence that men could identify which women were currently fecund when judging odours across different women.

Others have argued, however, that women's own partners may receive diagnostic information about fecundity since they can compare fertile window odours to odours from other days from the same woman [13]. Yet, when we constructed ROC curves for each rater by donor combination (thus measuring rater performance when they compared samples within the same women), discrimination performance was still very poor (figure 3). Thus, we found no evidence that men can reliably discriminate fertile window timing even when comparing samples within the same women.

An open question concerns whether discrimination performance may be higher when rating odours from familiar individuals. It is possible that long-term partners develop a representation of a given woman's range of odour variability that would help them calibrate to fecundity-associated changes, perhaps similar to the way that familiar males responded to facial cues of ovulatory timing more sensitively than did unfamiliar males in a sample of rhesus macaques [31]. If so, then high fecundity samples could be more salient to familiar than to unfamiliar raters, even if samples were rated under identical conditions. Whether women's own romantic partners can better discriminate their fertile window scents has not been tested. Note, however, that if only long-term romantic partners obtain sufficient sampling of odours to discriminate changes in fecundity, then ovulatory timing may already be concealed to a degree sufficient to encourage long-term pair bonding.

### (c) Hormonal predictors of odour attractiveness

At the within-woman timescale, we found no evidence that changes in hormones predict changes in odour attractiveness ratings. Thus, there is still no evidence for hormonal or other signals that may cause the elevated mean attractiveness ratings of late fertile window samples. Other signals associated with impending ovulation—including gonadotropins like LH, oxytocin [32], or testosterone [33]—could possibly affect odour attractiveness individually or via an unknown combination of signals. Alternatively, it is possible that measurement error helps to explain the lack of significant associations. Single salivary samples may be noisy measures of hormone production on a given day. Likewise, some prior research has reported evidence for time lags in the effects of hormones (e.g. [33]), but our sampling schedule of once every five days precluded tests of whether prior day hormone values predict odour attractiveness ratings. The physiological predictors of within-cycle changes in women's scents remains an open question for future research.

Null effects for associations between progesterone and odour attractiveness are especially noteworthy. Progesterone is highly elevated in humans only after the opportunity for conception has passed, as during pregnancy and in the luteal phase. Thus, an inhibitory effect of progesterone on odour attractiveness is predicted if perceivers can discriminate fertile window timing via odour cues (figure 1). Indeed, evidence supports such inhibitory effects of progesterone in nonhuman primates such as rhesus macaques [9,12]. Consistent with our findings, then, we propose phylogenetic changes somewhere in the evolutionary sequence to humans in which effects of progesterone on scent have been suppressed, thus weakening cues of low immediate fecundity and thereby promoting concealment of ovulatory timing. Further tests of this ‘progesterone suppression hypothesis' are necessary.

At the between-woman level of analysis, we did find that women with higher mean oestradiol were on average rated as smelling more attractive. Women who were 1 s.d. above the grand-mean in their average oestradiol concentration had odours rated approximately 0.5 s.d. above the grand-mean ratings (table 1), suggestive of a medium effect size. One prior study also reported a positive between-woman correlation between oestradiol and odour attractiveness when each was measured once per woman in the late follicular phase [23]. We suggest caution in the interpretation of this finding, however, for two reasons. First, our six hormone measurements were not collected on precisely the same cycle days across women, and this will have added noise into our estimations of mean oestradiol; studies that collect full cycles of hormone data would provide better tests of this relationship. Second, the effect is not necessarily causal. It is possible, for instance, that healthier women both smell better and produce higher oestradiol, on average, such that mean oestradiol correlates with but does not actually cause greater odour attractiveness. Manipulations of oestradiol would be necessary to rigorously test a causal relationship.

If the effect of oestradiol is causal, however, as in nonhuman species (e.g. [10]), then this raises interesting questions about the nature of hormone effects on odour attractiveness. A mean oestradiol effect in the absence of within-women effects suggests that oestradiol may have slower, longer-term effects on scents as opposed to changing odours on a shorter, day-to-day timescale (note, however, that measurement error may be lower for a mean of six samples than for any individual sample, which might partly explain larger effects for mean oestradiol). Oestradiol is elevated across cycle days in cycles with higher conception probability [20] and thus oestradiol-associated scent cues could provide information about current cycle fecundity to perceivers, more so than providing information specifically about fertile window timing (see [19]). Women in natural fertility populations spend long stretches of time in anovulatory states associated with events such as lactational amenorrhoea [34] and mechanisms in perceivers could in principle track between-cycle oestradiol variations in order to target behaviours such as mate guarding to time periods when partners are experiencing ovulatory cycles. These ideas could be tested by assessing whether within-women, between-cycle variability in mean oestradiol is positively associated with within-women, between-cycle variability in odour attractiveness.

Finally, independent of the effects of oestradiol, women in relationships were rated as smelling better than single women. This effect was not predicted *a priori* and was based on a relatively small number of total women in relationships. The effect thus warrants tests of replication but may also motivate future research to explain this relationship.

### (d) Limitations

Our findings apply only to ratings of scent attractiveness. It is possible that cues of ovulatory timing in women's odours affect other outcomes, such as men's reactive hormone responses ([35,36]; cf. [37]); further investigation of that question is necessary. Likewise, non-scent cues of ovulatory timing are possible, such as shifts in face, voice, or behavioural attractiveness ([4,38]; cf. [5,39]). Although mean differences across cycle regions have been detected for these other cues, the variability in these responses leads us to expect that discriminability of fertile window timing would also be very poor in such cases. Application of signal detection analyses to non-scent cues is an important direction for future research on the question of concealed ovulatory timing in humans. Finally, although our sampling of odours on six days per woman represented more frequent repeated sampling than in prior studies on this topic, a more ideal design might sample more women even more frequently in order to ensure that all cycle regions are represented evenly across a large sample of odour donors.

### (e) Conclusion

Our findings corroborate that peri-ovulatory odours in women do smell more attractive, on average, relative to scents from other cycle days, but our signal detection analyses show that discriminability of ovulatory timing is nonetheless very poor. At least with respect to odour attractiveness, then, the best available evidence suggests that human ovulatory timing is effectively concealed. This study provided the first test of hormonal predictors of within-women shifts in scent attractiveness, but we did not find any significant associations. We propose that the suppression of effects of progesterone on scent cues was an important evolutionary step in the concealment of human ovulatory timing. A positive association between women's mean oestradiol and their odour attractiveness leaves open the possibility that there are scent cues of overall cycle fecundity, and further tests of this possibility present intriguing directions for future research.

## Data Availability

All data are available from the Dryad Digital Repository: https://doi.org/10.5061/dryad.612jm6451 [40].
